# Genomic Sequencing to Detect Cross-Breeding Quality in Dogs: An Example Studying Disorders in Sexual Development

**DOI:** 10.3390/ijms251910763

**Published:** 2024-10-06

**Authors:** Luciana de Gennaro, Matteo Burgio, Giovanni Michele Lacalandra, Francesco Petronella, Alberto L’Abbate, Francesco Ravasini, Beniamino Trombetta, Annalisa Rizzo, Mario Ventura, Vincenzo Cicirelli

**Affiliations:** 1 Department of Biosciences, Biotechnology and Environment, University of Bari, Via Orabona 4, 70124 Bari, Italy; luciana.degennaro@uniba.it; 2 Department of Veterinary Medicine, University of Bari Aldo Moro, S.P. per Casamassima km. 3, 70010 Valenzano, Italy; matteo.burgio@uniba.it (M.B.); giovannimichele.lacalandra@uniba.it (G.M.L.); f.petronella7@studenti.uniba.it (F.P.); annalisa.rizzo@uniba.it (A.R.); 3 Institute of Biomembranes, Bioenergetics, and Molecular Biotechnology (IBIOM), 70125 Bari, Italy; a.labbate@ibiom.cnr.it; 4 Department of Biology and Biotechnologies ‘Charles Darwin’, Sapienza University of Rome, 00185 Rome, Italy; francesco.ravasini@uniroma1.it (F.R.); beniamino.trombetta@uniroma1.it (B.T.)

**Keywords:** dogs, genomics, French Bulldog, breed, run of homozygosity

## Abstract

Disorders of sexual development (DSDs) in dogs, similar to humans, arise from genetic mutations, gonadal differentiation, or phenotypic sex development. The French Bulldog, a breed that has seen a surge in popularity and demand, has also shown a marked increase in DSD incidence. This study aims to characterize the genetic underpinnings of DSDs in a French Bulldog named *Brutus*, exhibiting ambiguous genitalia and internal sexual anatomy, and to explore the impact of breeding practices on genetic diversity within the breed. We utilized a comprehensive approach combining conventional cytogenetics, molecular techniques, and deep sequencing to investigate the genetic profile of *Brutus*. The sequence data were compared to three other male French Bulldogs’ genome sequences with typical reproductive anatomy, including *Brutus*’s father and the canine reference genome (CanFam6). We found a Robertsonian fusion involving chromosome 23 previously reported in dogs as a causative mutation responsible for sex reversal syndrome. Our findings revealed a 22% mosaicism (78,XX/77,XX), the absence of the sex-determining region (*SRY*) gene, and the presence of 43 unique Single Nucleotide Variants (SNVs) not inherited from the father. Notably, the run of homozygosity (ROH) analysis showed *Brutus* has a higher number of homozygous segments compared to other Bulldogs, with a total length of these fragments 50% greater than the average, strongly suggesting this dog is the product of the mating between siblings. Although no direct causative genes for the DSD phenotype were identified, four candidate loci warrant further investigation. Our study highlighted the need for a better annotated and curated reference dog genome to define genes causative of any specific phenotype, suggests a potential genetic basis for the DSD phenotype in dogs, and underscores the consequences of uncontrolled breeding practices in French Bulldogs. These findings highlight the importance of implementing strategic genetic management to preserve genetic health and diversity in canine populations.

## 1. Introduction

Physiological sexual differentiation in humans, as in dogs, depends on genetic determinants, gonadal differentiation, and the development of the phenotypic sex. An irregularity in any of these three steps can lead to disorders of sexual development (DSDs) [1,2]. DSDs have been extensively studied in dogs, and thanks to their similarity with humans, they have been used to highlight molecular mechanisms underlying the occurrence of DSDs [3,4]. These conditions are considered rare in the canine species, and their variability in phenotypic expression with ambiguous external genitalia makes the diagnosis and a unique classification of intersex conditions difficult [3]. Following the human classification [5], there are three major categories: [1] DSD caused by an abnormal set or structure of sex chromosomes, called sex chromosome DSD; [2] DSD with a regular female set of sex chromosomes, called XX DSD, and [3] DSD with a standard complement of male chromosomes, XY DSD [2,4]. The first group includes all those subjects whose karyotype shows numerical variations of chromosomes, such as monosomies of the X chromosome, trisomies XXX or XXY, and chimerism phenomena in which subjects show cells with both XX and XY sex chromosomes at the same time.

Despite numerous studies, the chromosomal background of these anomalies in dogs remains to be seen [6] mainly because karyotyping in dogs presents a significant challenge due to the high chromosome count (2n = 78) and the acrocentric nature of all predominantly small autosomes [7]. Only the sex chromosomes are metacentric and easily distinguishable from the autosomes. Easily identifiable mutations, such as those involving sex chromosome aneuploidies primarily associated with DSDs, are X monosomy [8], X trisomy [9], and XXY sex chromosomes [10].

Despite extensive analyses, no causative point mutations have been found in specific candidate genes to explain these disorders. However, a few studies have shown a link between XX DSDs and mutations such as copy number variations, duplications, and substitutions in Sry-box containing gene nine or Sry-like HMG box (*SOX9*) gene, an essential gene having a role in testis induction in vertebrates [11]. The SRY gene, essential for male development, provides further insights into DSD types by working with the *SOX9* gene to produce testosterone and anti-Müllerian hormone (*AMH*), leading to male genitalia formation [2,12]. Recent research has suggested an association between peptidyl arginine deiminase 6 (*PADI6*) gene variants and XX DSDs in certain dog breeds, highlighting the complex and breed-specific nature of genetic variations in DSD phenotypes [13]. DSDs have been detected in more than 40 dog breeds, with an increased incidence in American Cocker Spaniels, English Cocker Spaniels, Kerry Blue Terriers [14], American Staffordshire Terriers, and French Bulldogs [2]. XX DSDs are the most frequently seen disorders, with an increase in incidence in recent years in the French Bulldog. This is probably due to uncontrolled mating that responds to the primarily increased demand in the market. French Bulldogs represent the most popular breed in the United Kingdom in 2018 [2,15,16]. Recent data on DSDs in dogs have highlighted the significant increase over the years of this disorder in the French Bulldog breed [2]. This increase in cases clearly outlines a stable growth trend that suggests the need to investigate the underlying causes of this process for a clearer view of the pathology.

For this reason, we used a multipronged approach to deeply characterize an 11-month-old French Bulldog named *Brutus*, with ambiguous external genitalia consisting of a vulva containing a penis-like structure and internal genitalia displaying two testicles connected to a complete uterus. We deeply investigated the genetic profile of the phenotypic condition associated with our sample using conventional and molecular cytogenetics, deep sequencing, molecular biology techniques, and three other healthy dogs. We performed the run of homozygosity (ROH) analysis, revealing that *Brutus* has the highest number of homozygous segments than the controls, leading to a total length of these homozygous fragments 50% higher than the average of other individuals from different breeders. Although this work did not allow us to find a direct correlation with the DSD phenotype, we were able to highlight human R-spondin1 (*RSPO1*), *SOX9*, and steroid 5 alpha-reductase 2 (*SRD5A2*) as candidate genes that need further study. Noteworthily, the sequencing approach highlighted how the uncontrolled cross-breeding carried out by Bulldog breeders currently has genotypic and, consequently, phenotypic effects. This underscores the need for such an approach to plan genetic management in canine populations properly.

## 2. Results

**Figure 1:**
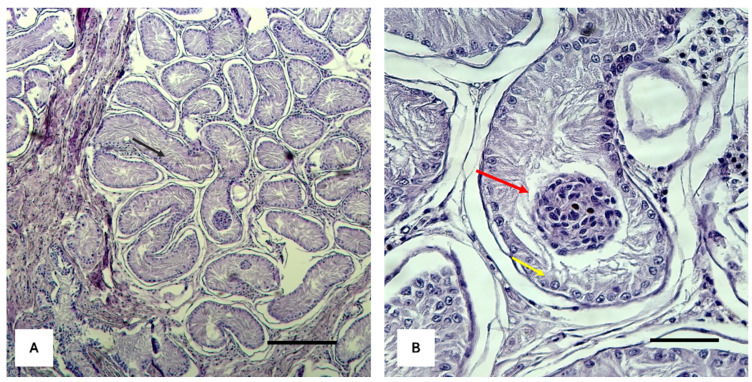
Histological analysis of the testicular parenchyma removed during laparoscopic surgery. The images display tissue sections stained with hematoxylin and eosin (H&E), revealing the structural and cellular characteristics of the testicular tissue. (**A**) Seminiferous tubule with undeveloped germinal epithelium (black arrow); (**B**) Sertoli cells with nucleated nuclei (yellow arrow); immature germ cell aggregates in the lumen (red arrow) (scale bar = 50 μm).

At the clinical examination, *Brutus* showed a generally good state of nutrition. The external genitalia showed a normal vulva in terms of position, shape, and size; the vulvar lips showed inflammation and excoriation of the skin part caused by the patient’s continuous licking of the area. The labia could not close the vulvar opening due to the presence and protrusion of the penile structure. The penis, containing the penile bone, showed a dry, inflamed, and eroded surface due to the lack of skin covering and protection. There were no abnormalities in the course of the internal urethra and the external part of the organ connected to the external sphincter located on the dorsal face of the body of the penis. The internal genitalia, investigated by second-level diagnostic means (ultrasound and computed tomography), showed two compact intra-abdominal parenchymatous structures connected to two cavitary organs referred to as uterine horns (Appendix A). Following laparoscopic surgery to remove the intra-abdominal organs, histological analysis showed testicular parenchyma presenting seminiferous tubules with undeveloped germinal epithelium and Sertoli cells with nucleated nuclei and no mature spermatozoa (Figure 1).

**Figure 2:**
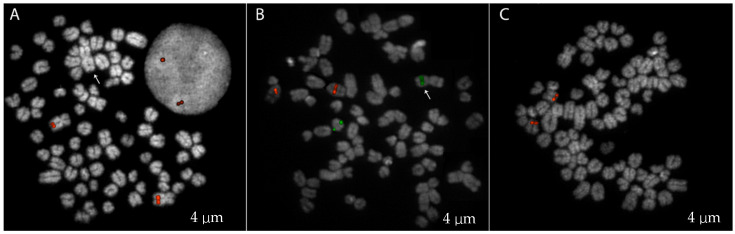
FISH analysis on *Brutus* using different probes. (**A**) FISH with an X-specific probe (CH82-201N14), showing both metaphase chromosomes and interphase nuclei. The arrow indicates the additional metacentric chromosome (**B**) FISH using probes for chromosome 5 (CH82-509B23; red) and chromosome 23 (CH82-253P13; green). The arrow shows the translocated metacentric chromosome (**C**) FISH with a SOX9-specific probe (CH82-26I8).

Additionally, seven slides with metaphase preparations were obtained from the blood of *Brutus*, stained with 4′,6-diamidino-2-phenylindole (DAPI), and visualized under a fluorescence microscope to evaluate the chromosomal structure and perform a classical karyotype analysis. We observed 82 metaphases obtained from the lymphocyte culture. We karyotyped 10 metaphases using standard karyotype as a reference [7] and showed the presence of two metacentric X chromosomes and the absence of the Y chromosome. To confirm the identity of these large submetacentric chromosomes present in all metaphases, we performed a FISH experiment using a dog-specific probe (CH82-201N14, chrX:92883400-93054893 CamFam6 reference genome) (Figure 2A).

The chromosome count was performed, and in 18 out of 82 metaphase spreads, we observed 77 chromosomes with an additional non-acrocentric chromosome lacking the chromosome X-specific signal (Figure 2A). A FISH experiment using two specific probes for chromosomes 5 and 23, CH82-509B23 and CH82-253P13, respectively, was performed to check whether the additional metacentric chromosome found in some metaphases was the result of a Robertsonian translocation like the one previously reported in another case of sex reversal dog [17]. Of note was that we detected the presence of a chromosome 23 probe on the additional metacentric, showing a different Robertsonian fusion than the previously reported (Figure 2B).

The conventional and molecular cytogenetic analysis was also performed on the other three healthy control dogs analyzed, including *Brutus*’s father, and in all cases, a normal 78, XY karyotype was found (Figure S1).

To investigate the presence of the *SRY* gene, an additional FISH experiment with a human-specific probe (RP11-400O10) for the *SRY* gene was used on the four dogs we were testing. None of the experiments was successful, likely due to the high divergence between the target (dog genome) and probe (human source). We further investigate the presence of the *SRY* gene in *Brutus* by performing a PCR using primers canine-SRY-specific [18]. A 271 bp expected amplification product was obtained only in *Brutus*’s father and in the other two controls (*Tauro* and *Bufalo*), confirming what was already karyotypically hypothesized in which our case with DSD is also SRY negative (Figure S2).

The *SOX9* gene, which is often associated with DSDs in humans [19], was also investigated using the CH82-26I8 probe specific for this region on chromosome 9 [20]; however, no significant deletion or duplication was highlighted (Figure 2C). We analyzed this gene at the sequence level to confirm this result, and no mutations that jeopardized gene function were found.

The alignment and variant calling of the sequenced genomes were performed using CanFam6 as the reference genome. The decision to use the genome of the Boxer breed as a reference is supported by phylogenetic [21] and cluster [22] analyses in the literature, which demonstrated the highest correlation between this genome and the French Bulldog. This approach ensures that the reference genome is closely related to the genetic background of our samples, thereby increasing the accuracy and relevance of the alignment and variant calling results. Following the annotation performed on the variant calling output (see Section 4), we identified a total of 43 SNVs that are unique to the *Brutus* genome. All these variants identified have been classified, after SNPeff annotation, as having a putative impact level of “modifier” (Table S1). In total, twenty of the forty-three identified SNVs are on autosomal chromosomes [2,8,14,18,23,24], nine on the X chromosome, and fourteen on mitochondrial DNA. These variants could be significant for DSD phenotype since they were never detected in the sequence of other healthy Bulldogs. The alleles detected in the other three Bulldogs could represent specific breed variations.

To assess the homozygosity patterns within our samples, we conducted a run of homozygosity (ROH) analysis, focusing on genomic segments exceeding 1000 kb and containing at least 100 SNPs. Our study revealed that the healthy dogs (*Tauro*, *Bufalo*, and *Brutus*’s father) exhibited an average of 159 ROH segments, with a mean total length of 303,390 kb. In contrast, *Brutus* largely deviates from these average values; the ROH analysis for *Brutus* identified 298 homozygous segments with a total length (681,002 kb) exceeding twice the average size.

Table S2 details the localization, number of SNPs, and percentage of homozygosity for each ROH fragment.

Considering the total length of autosomal chromosomes, which is 2,212,284 kb, the values of F_ROH (fraction of ROH) for *Brutus* were markedly higher than those observed in the other samples and higher than the expected value between the two siblings (0.25) (Table 1).

Within the 298 *Brutus* ROH segments, 593 genes were identified (according to the UCSC RefseqCurated database for CanFam4; Table S3). Of the 593 genes found, only 508 were recognized and analyzed by the ToppGene portal. The excluded genes (“not found” in column 2 of Table S3) were predominantly miRNAs and olfactory receptor genes. The results obtained from ToppGene were categorized into “Biological Process” (Table S4), “Mouse Phenotype” (Table S5), “Disease” (Table S6), and “PubMed” (Table S7). Within each category, we searched for the keywords “Development”, “Sex”, and “hermaphroditism/hermaphroditic” to search for the most significant results associated with our phenotype of interest. We found fourteen in PubMed, twelve in Biological Process, three in Mouse Phenotype, and one in Disease (items highlighted in red in Tables S4–S7), involving 81 genes (Table S8). Among these, SRD5A2, RSPO1, and SOX9 genes merit further investigation in dogs, as they have already been associated with our phenotype of interest in other species, according to PubMed publications.

## 3. Discussion

Using a multipronged molecular approach, we performed a comprehensive genetic analysis on a French Bulldog named *Brutus* with DSD and found significant information on this case’s chromosomal and genetic characteristics. Karyotypic analysis and molecular cytogenetics revealed the presence of two metacentric X chromosomes and the absence of the Y chromosome. Using a specific probe targeting the X chromosome confirmed the sex chromosome configuration in *Brutus*. We confirmed the absence of the sex-determining region Y (*SRY*) in *Brutus* by PCR using canine-specific *SRY* primers. These findings are consistent with similar cases of sex reversal in dogs that are genetically female, 78,XX [15].

Additionally, in 82 metaphases analyzed, we detected approximately 22% 78,XX/77,XX mosaicism. Using several FISH experiments, we identified an additional metacentric in the metaphase spread with 77 chromosomes due to a Robertsonian fusion between chromosome 23 and an unidentified chromosome. Unlike a previously reported DSD case in which the translocation involved chromosomes 5 and 23 [17], our results did not show the involvement of chromosome 5, suggesting a different chromosomal partner in our translocation event. This highlights the genetic complexity of the DSD phenotype and supports the possibility that the development of DSDs, although not related to a specific Robertsonian translocation, could somehow be associated with rearrangements of chromosome 23 that could potentially affect the regulatory sequences of genes located on chromosome 23 (*FOXL2* and *CTNNB1*) involved in reproductive development [17].

The absence of the SRY gene in *Brutus*, confirmed by PCR, identified this dog as an *SRY*-negative XX DSD phenotype. The investigation of the *SOX9* gene, responsible for the DSD phenotype in humans, revealed no significant structural variations such as deletions or duplications, suggesting that other genetic or epigenetic factors might contribute to the observed DSD phenotype in *Brutus*. Previous studies have associated the virilization of XX dogs with the SOX9 gene copy number and increased copy number in the CNV region upstream of this gene [24]; however, our study did not detect these associations. This implies that the DSD phenotype in *Brutus* may involve alternative loci, mechanisms, and genetic interactions.

The genomic sequencing of our French Bulldog and the other three control dogs, including his father, has provided more profound insights into the genetic underpinnings of this DSD phenotype. Our analysis identified 43 SNVs unique to *Brutus*. Although these variants are classified with a modifier impact—suggesting they may not directly affect protein function—they could represent non-coding or regulatory variations potentially influencing gene expression and contributing to the observed phenotype. Notably, eight SNVs are clustered within an 800 bp region on the X chromosome, specifically upstream of the spermiR sequence. SpermiRs are microRNAs critical for spermatogenesis and exhibit high and testis-specific expression patterns conserved across several mammalian species [25]. This localization suggests that, while each SNP individually may have a minimal effect, their cumulative influence could be significant, potentially affecting spermiR’s gene regulation and, consequently, impacting the expression of downstream genes involved in fertility [25]. This would explain the histological results showing the lack of mature spermatozoa, the presence of an incompletely developed germinative epithelium, and Sertoli cells with nuclear alterations. Other significant discoveries made by ROH analysis provided further insights into the genetic architecture of *Brutus* compared to healthy controls. *Brutus* showed higher ROH segments and total ROH length, indicating a higher degree of homozygosity. This high level of homozygosity suggests significant inbreeding, contributing to the accumulation of homozygous deleterious alleles, which may be responsible for the observed phenotype.

The high F_ROH value, considering the total length of the autosomal chromosomes, suggests inbreeding, which may have exposed recessive alleles that contribute to the DSD phenotype. Inbreeding, while helpful in maintaining breed purity, can significantly impact genetic health by increasing homozygosity, which can correct advantageous and deleterious traits within the population [26,27,28]. Genomic inbreeding coefficients, which measure the proportion of an individual’s DNA inherited from recent common ancestors, provide a direct measure of inbreeding and are more accurate than pedigree-based estimates. This study shows that *Brutus* has a higher genomic inbreeding coefficient than control dogs, indicating a recent inbreeding event, most likely by mating between siblings. Typically, the inbreeding coefficient for an individual born from a sibling mating is around 0.25. However, in our analysis, *Brutus* exhibited an F_ROH of 0.31, higher than expected. This higher value is likely because the common ancestors of *Brutus*’s parents were not fully allozygous, indicating a certain level of autozygosity in the breed. This is supported by the finding that all three individuals analyzed have comparable inbreeding coefficient values. To contextualize this value, we calculated the average inbreeding level of the breed by averaging the F_ROH of three normal genomes. We then estimated the theoretical inbreeding coefficient for an individual born from a sibling mating, taking into account the average value of the three normal individuals as a baseline level of inbreeding within the population. Interestingly, our calculations yielded a theoretical value of 0.28 (0.29 when using the F_ROH value of the father of *Brutus*), closely matching the empirically observed value. These findings support the hypothesis that *Brutus* is the offspring of related parents, precisely two siblings.

The findings from our ToppGene analysis provide valuable insights into the genetic underpinnings of the phenotype observed in *Brutus*. By examining the genes present within the ROHs in *Brutus*, we identified 81 genes that are probably related to our phenotype of interest. The presence of these genes in a homozygous state, likely due to recent inbreeding, could be implicated in a complex mechanism involving multiple pathways, all associated with sex development and hermaphroditism. Inbreeding can lead to an increased homozygosity of deleterious alleles, which can unmask recessive traits and disrupt normal developmental processes. Particularly noteworthy among these genes are *SRD5A2*, *RSPO1*, and *SOX9*. These genes have been highlighted in prior PubMed publications as associated with similar phenotypes in other species [26,29,30]. Identifying these genes in our canine case suggests potential parallels in the genetic mechanisms underlying sexual development and hermaphroditism across species. This cross-species relevance underscores the importance of further investigating these genes in dogs.

In summary, our study underscores the complexity of disorders of sexual development (DSDs) in dogs, highlighting the combination of chromosomal anomalies, absence of critical sex-determining genes, and unique genetic variants as underlying factors. This complexity is further compounded by the dual nature of inbreeding in breed management. While it aids in preserving breed-specific traits, it simultaneously elevates the risk of phenotypic abnormalities such as DSDs. Future studies should focus on characterizing the specific mutations and their phenotypic consequences in the canine model, providing deeper insights into the genetic regulation of sexual development and contributing to our understanding of similar disorders in other species. Additionally, understanding the genetic basis of these conditions could lead to improved diagnostic tools and inform strategies for managing or preventing such phenotypes in dog populations. Furthermore, future research should explore the regulatory mechanisms and potential epigenetic modifications that could elucidate the pathways involved in canine DSDs and consider the balance between maintaining breed purity and genetic health. Such investigations could ultimately pave the way for more effective approaches to breeding management and disease prevention in dogs.

## 4. Materials and Methods

### 4.1. Clinical Characterization of Brutus

The patient, *Brutus*, a French Bulldog, 11 months old and weighing 9 kg with a height of about 30 cm at the shoulder, at the general objective examination, was in good health and nutrition with a body conditions score (BCS) of 5/9. Clinical examination showed a compact, muscular body with a deep, broad chest. It had a typically brachycephalic head, flattened face, short muzzle, and flattened nose, and a short, smooth, blue-colored coat with a wide white patch on the chest. The tail is naturally short and slightly coiled—proportionate and muscular limbs with solid posture. Observable behavior during clinical examinations is lively and curious. A unique examination of the external genital area showed a reddened, dry, and lesioned vulva from which a penis was approximately 5 cm long and 0.5 cm wide with an external urethral orifice and an opening in the dorsal position. In its anamnesis, *Brutus* had signs of typical male dog urination.

### 4.2. FISH Experiments

Canine lymphocytes derived from *Brutus*’s peripheral blood sample were stimulated with phytohemagglutinin (PHA) and used to prepare the metaphase spreads with the standard procedures [31].

Cytogenetic analyses were performed by fixing metaphase spreads onto slides and then incubated at 90 °C for 1 h 30′ to contribute to sample fixation and dehydration. The 0.005% pepsin/HCl 0.01 M treatments were performed to eliminate cytoplasm proteins for better hybridization rates. Subsequent treatments in PBS 1×, MgCl_2_ 0.5 M, 8% paraformaldehyde, and 70%/90%/absolute alcohols allowed proper stabilization, fixation, and dehydration of metaphases and nuclei DNA molecules. In situ fluorescence hybridization (FISH) experiments were performed on nuclei and metaphases using probes from a female canine BAC CHORI-82 library (https://bacpacresources.org/library.php?id=253 (accessed on 27 September 2024)) and human RP11 BAC library (Table 2).

DNA extraction from selected BACs was performed using the Quantum Prep Plasmid Miniprep Kit (Bio-Rad, Hercules, CA, USA). FISH experiments were essentially performed as previously described [32]: two hundred nanograms of the DNA probe, labeled by nick-translation with Cy3-dUTP or fluorescein-dUTP, were precipitated by ion-exchange alcohol precipitation with canine Cot1 DNA and finally denatured for 2 min at 70 °C and hybridized at 37 °C overnight. Post-hybridization washing was at 60 °C in 0.1× SSC (three times, high stringency). The hybridization with the human probe was performed at 72 °C for 2 min, and post-hybridization washes were carried out under low stringency conditions, precisely three washes at 38 °C with 2× SSC. The slide was then stained with DAPI, producing a Q-banding pattern. The fluorescence signals coming from Cy3, Fluorescein, and DAPI were detected separately with specific filters using a Leica DMRXA epifluorescence microscope equipped with a cooled CCD camera (Teledyne Princeton Instruments, Acton, MA, USA) and recorded as grayscale images. Finally, Adobe Photoshop™ software (2024) was used for image pseudo-colorization and merging of the acquired images.

### 4.3. Genomic Extraction

Genomic DNA of all the samples was extracted, starting from blood samples with the DNA Blood mini-kit (Qiagen Inc., Germantown, MD, USA), following the manufacturer’s protocol. The Nanodrop spectrophotometer measured the DNA concentrations.

### 4.4. PCR

To investigate the presence of SRY in *Brutus*, a PCR using primers specific for canine SRY (Forward: 5′ AAGGCCACGGCACAGAAAAGTCAC and Reverse: 5′ AAGAAGCGTCAGCGGACATCTGTG) from [18] was performed using iProof HF Master Mix (Bio-Rad, Hercules, CA, USA). Amplification was carried out in 25 μL reactions with 1 × PCR MasterMix, 0.5 μm forward and reverse primers, and 50 ng of genomic DNA. The reaction was then cycled with the following conditions: initial denaturation at 98 °C for 3 min, then 30 cycles at 98 °C for 10 s, 70 °C for 30 s, and 72 °C for 20 s; final extension was at 72 °C for 7 min.

### 4.5. Sequencing Analysis: Variant Calling and ROH Estimation

The extracted DNA was sent to the CD Genomics Institute for DNA QC, Library Preparation (PE150), and NovaSeq6000 S4 Sequencing (30×) (illumina Inc., San Diego, CA, USA).

Raw reads were aligned to the most recent dog reference genome available (Dog10K_boxer_Tasha/CanFam6, October 2020) using the BWA-MEM algorithm (defaut version 0.7.17) with the default parameters (https://bio-bwa.sourceforge.net (accessed on 27 September 2024)), and we removed PCR duplicates using the Picard MarkDuplicates tool (version 3.1.1, http://broadinstitute.github.io/picard/ (accessed on 27 September 2024)). Variant calling analysis was performed using GATK (version 4.3.0.0) [23] with “HaplotypeCaller” and “-ERC GVCF”. After combining (“CombineGVCFs”) the gVCFs of the individual samples, the merged file was then genotyped (“GenotypeGVCFs”) and filtered only for SNP-type variants (“SelectVariants” and “--select-type-to-include SNP” options). Using “VariantFiltration”of GATK with the “--filter-expression” option, we discarded SNVs with a “DP” value (read depth) lower than the 75th percentile of the DP values, and the SNPs with QD < 2.0, FS > 60.0, MQ < 56.0, SOR > 3.0, MQRankSum < −12.5, ReadPosRankSum < −8.0.

By evaluating the positions in which there was a call in all the samples, only the SNVs in which a specific variant is present only in *Brutus* and never, even in heterozygosity, in the other samples were selected.

We downloaded, from the UCSC Genome Browser, the complete SNP database (dbSNPs), based on the “CanFam4” genome release (https://genome.ucsc.edu/cgi-bin/hgTables?hgsid=2314426054_ivCEAdUZP0HdC5ZSWlzUb5F20Rzu (accessed on 27 September 2024)), and we converted all SNP positions to the “CanFam6” release, the one used for generating the BAM files. We used the converted dbSNPs to filter out common SNPs in our samples. Finally, SnpEff (v5.2) (https://pcingola.github.io/SnpEff/ (accessed on 27 September 2024)) software was used to annotate the list of variants that had been retained.

To quantify genomic inbreeding, we measured the proportion of an individual’s DNA inherited from recent common ancestors, providing an estimate of the percentage of the individual’s inbred genome. Runs of homozygosity were identified using the --homozyg function in PLINK v1.9, considering only genomic segments extending more than 1000 kb (--homozyg-kb) and with at least 100 SNPs (--homozyg-snp). The resulting ROH segments were analyzed to determine the genomic inbreeding coefficient (F_ROH), representing the proportion of the autosomal genome (total length of autosomal chromosomes = 2,212,284 kb) covered by these segments.

After analyzing the ROHs, we identified the genes in *Brutus*’s homozygous segments. The analysis was performed in R (version 4.2.2, R Core Team, 2022) using the overlap function from the data—table library, following a liftover to CanFam4 to utilize the most comprehensive RefSeq track available. We used CanFam4 instead of the most recent release, CanFam6, because of the highest accuracy in gene annotation of the former. The obtained gene list was analyzed using the ToppGene portal (https://toppgene.cchmc.org/ (accessed on 27 September 2024)), as described in [33], a comprehensive platform for gene list enrichment analysis and candidate gene prioritization based on functional annotations and protein interaction networks. The ToppFun function was explicitly utilized to detect functional enrichment of genes based on various ontologies (GO, pathway), mouse phenotype, literature cocitation, and other features.

## Figures and Tables

**Table 1:** ROH analysis results for the healthy control dogs (*Tauro*, *Bufalo*, and *Brutus*’s father) and *Brutus*. Each column presents the identifier for each dog (SAMPLE), the total count of homozygous segments identified (NSEG), the cumulative length of all ROH segments per sample (KB), the average length, in kb, of ROH segments (KBAVG), and the average inbreeding coefficient (F_ROH).

SAMPLE	NSEG	KB	KBAVG	F_ROH
*Brutus*	298	681,002	2285.24	0.308
*Bufalo*	153	299,507	1957.56	0.135
FatherB	170	318,511	1873.6	0.144
*Tauro*	154	292,152	1897.09	0.132

**Table 2:** Probes used for the FISH experiments. The mapping for the BAC probes derived from the canine CH82 library is based on the CanFam6 reference genome, while the BAC probe from the human RP11 library is mapped to the hg38 reference genome.

PROBE	MAPPING
CH82-201N14	chrX:92883400-93054893
CH82-509B23	chr5:30780182-30905463
CH82-253P13	chr23:26062226-26251145
CH82-26I8	chr9:9505916-9667419
RP11-400O10	chrY:2724275-2921156

**Figure A1:**
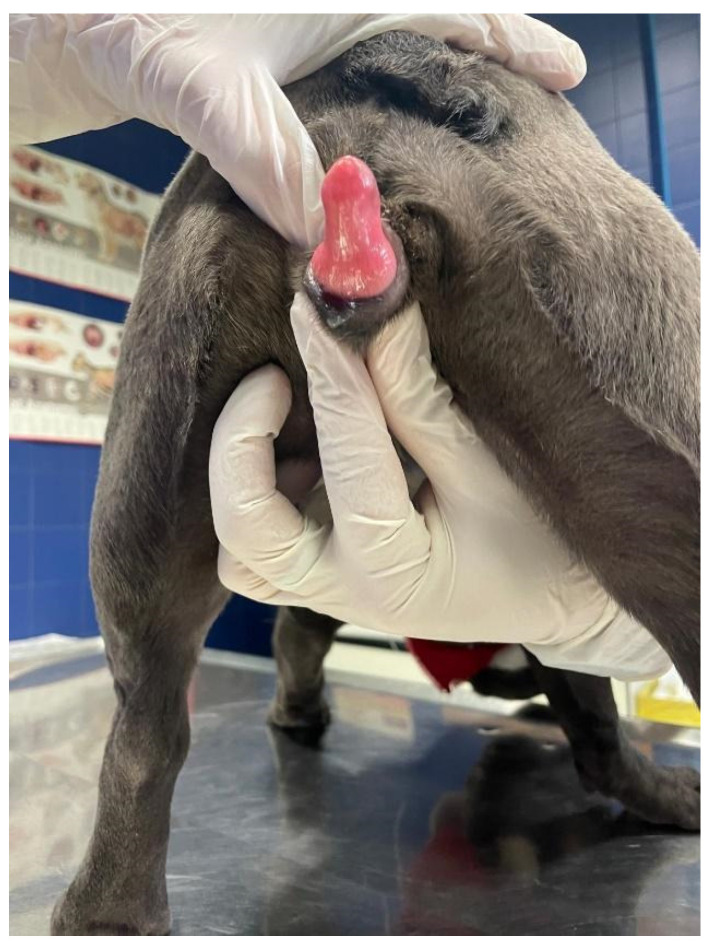
Caudo-ventral vision of penis-like structure.

**Figure A2:**
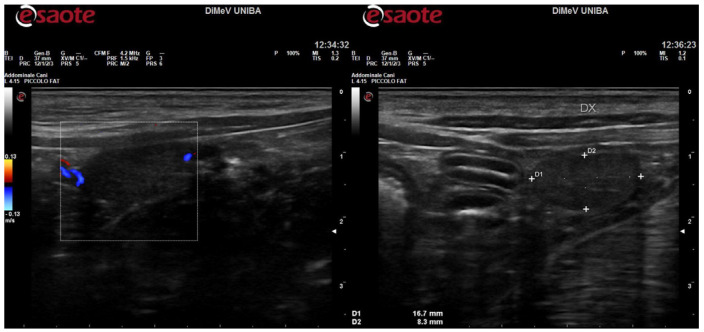
Ecographic study of the testicle-like abdominal structure using color Doppler and in B-Mode.

**Figure A3:**
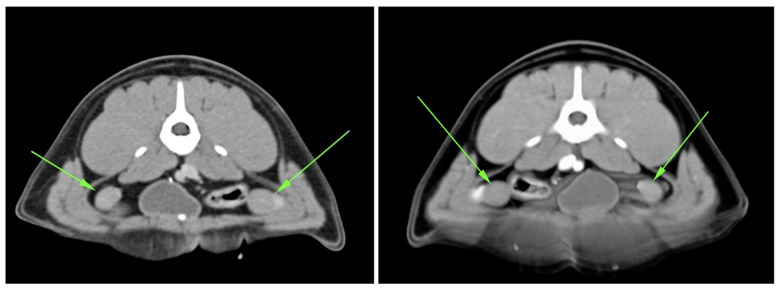
CT images showing intra-abdominal testicular structures (green arrows).

**Figure A4:**
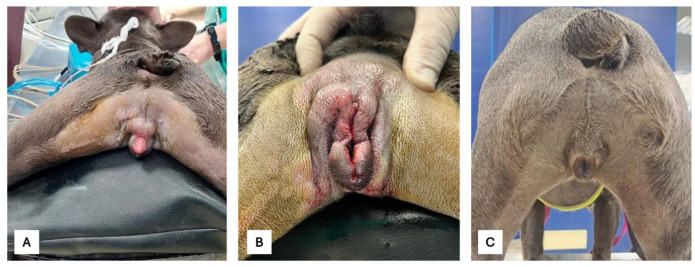
The patient in pre-operative conditions (**A**), after 3 h (**B**), and after 6 months from the surgery (**C**).

## Data Availability

The data supporting this study’s findings are available from the corresponding authors upon reasonable request.

## Supplementary Materials

The following supporting information can be downloaded at: https://www.mdpi.com/article/10.3390/ijms251910763/s1.

## Abbreviations

*AMH*	anti-Müllerian hormone
CNV	copy number variation
*CTNNB1*	catenin beta 1
DAPI	4′,6-diamidino-2-phenylindole
DSD	disorders of sexual development
FISH	fluorescence in situ hybridization
*FOXL2*	forkhead box L2
HMG	high-mobility group
NSEG	number of segments
*PADI6*	peptidyl arginine deiminase 6
*RBMYA1*	RNA binding motif protein Y-linked family 1 member A1
ROH	run of homozigosity
*RSPO1*	R-spondin1
SNP	single nucleotide polymorphism
SNVs	single nucleotide variants
*SOX9*	Sry-box containing gene 9 or Sry-like HMG box
SpermiR	spermatogenesis-related miRNAs
*SRD5A2*	steroid 5 alpha-reductase 2
*SRY*	sex-determining region

## Appendix A

Case description.

A French Bulldog called *Brutus* was referred to the Obstetrical Clinic Section of the Department of Veterinary Medicine at the University of Bari. The patient, 11 months old and weighing 9 kg at the general objective examination, was in good health and nutrition with a body conditions score (BCS) of 5/9. The owner considered his dog to be female and complained of continuous and obsessive licking of the peri-vulvar area due to a protruding “vaginal mass”. A special examination of the external genital area showed a reddened, dry, and lesioned vulva from which a penis was approximately 5 cm long and 0.5 cm wide with an external urethral orifice and an opening in the dorsal position (Figure A1).

The clinical examination was completed with a blood draw performed using Vacutainer tubes with EDTA and serum collection. The sample, maintained at a constant temperature of 4 °C, was sent and processed within 4 h from collection to preserve cell viability. General blood tests (blood count, biochemistry, and plasma protein electrophoresis) were performed (Table A1 and Table A2).

**Table A1:** Blood tests analysis.

Parameters	Sample	Reference Range	Parameters	Sample	Reference Range
**RBC (milioni/µL)**	**6.39**	**5.50–8.00**	**WBC (×1000/µL)**	**11.8**	**6.0–14.0**
**HGB (g/dL)**	**16.0**	14.0–19.5	**Correct counting WBC (×1000/µL)**	11.8	6.0–14
**HCT (%)**	**50.4**	38.0–54.0	**Myelocytes (/µL)**	0	0
**MCV (fL)**	**78.9**	60.0–73.0	**Metamyelocytes (/µL)**	0	0
**MCH (pg)**	**25.0**	21.0–27.0	**Band neutrophils (/µL)**	0	0–300
**MCHC (g/dL)**	**31.7**	33.0–37.0	**Segmented neutrophils (/µL)**	7316	3500–9300
**CHCM (g/dL)**	**31.6**	32.0–37.0	**Linfocytes (/µL)**	3304	1200–3200
**CH (pg)**	**24.8**	21.5– 25.7	**Monocytes (/µL)**	708	200–1200
**CHDW (pg)**	**2.76**	3.18–3.80	**Eosinophils (/µL)**	472	100–800
**RDW (%)**	**12.6**	12.0–16.0	**Basophils (/µL)**	0	0–100
**HDW (g/dL)**	**1.57**	1.50–2.50	**Emoparasites**	NEG.	NEG.
**NRBC/100 WBC**	**0**	0			
**PLT (1000/µL)**	**203**	180–450			
**MPV (fL)**	**10.8**	8.5–14.5			
**PCT (%)**	**0.22**	0.20–0.50			
**PDW (%)**	**46.8**	53.0–70.0			
**MPC (g/dL)**	**16.0**	18.0–24.0			
**MPM (pg)**	**1.6**	1.5–2.45			
**Large PLT (1000/µL)**	**67**	7–50			

**Table A2:** Biochemical analysis.

Parameters	Sample	Reference Range	Parameters	Sample	Reference Range
**CPK (IU/L)**	**329**	**30–160**	**Calcio (mg/dL)**	**10.5**	**9.0–11.5**
**AST (IU/L)**	**56**	18–50	**Calcio corretto (mg/dL)**	**10.4**	9.0–11.5
**ALT (IU/L)**	**110**	20–75	**Fosforo (mg/dL)**	**7.5**	2.8–4.7
**ALP (IU/L)**	**66**	20–160	**Magnesio (mg/dL)**	**2.3**	1.4–2.1
**GGT (IU/L)**	**1.4**	2.6–7.4	**Sodio (mEq/L)**	**145**	140–152
**Colinesterasi (IU/L)**	**6689**	3350–7250	**Potassio (mEq/L)**	**4.7**	4.0–5.3
**Bilirubina Totale (mg/dL)**	**0.09**	0.10–0.30	**Rapporto Na/K**	**30.9**	28.0–37.0
**Proteine Totali (g/dL)**	**6.3**	5.7–7.3	**Cloro (mEq/L)**	**107**	105–115
**Albumine (g/dL)**	**3.3**	2.8–3.7	**Cloro corretto (mEq/L)**	**107.7**	109–115
**Globuline (g/dL)**	**3.0**	2.8–3.9	**HCO-3 (mmol/L)**	**20.3**	15.8–22.3
**Rapporto A/G**	**1.10**	0.70–1.30	**Divario Anionico**	**22.4**	18.5–29.0
**Colesterolo (mg/dL)**	**255**	130–330	**Osmol. sier. mis. (mOsm)**		292–310
**Trigliceridi (mg/dL)**	**25**	30–130	**Osmol. sier. calc. (mOsm)**	**282**	277–297
**Amilasi (IU/L)**	**749**	411–1522	**Div. Osmolale**		18–28
**Lipasi (IU/L)**	**281**	77–585	**Ferro totale (µg/dL)**	**228**	90–230
**Urea (mg/dL)**	**26**	15–50	**UIBC (µg/dL)**	**186**	180–350
**Creatinina (mg/dL)**	**1.13**	0.70–1.40	**TIBC (µg/dL)**	**414**	300–500
**Glucosio (mg/dL)**	**91**	70–130	**Saturazione (%)**	**55.1**	20–60
**Lipasi DGGR (UI/L)**	**65**	13–117	**Prot. C Reattiva (mg/dL)**	**0.03**	0.01–0.45

Additionally, imaging tests were performed using an Esaote MyLab Alpha device (Figure A2) and computed tomography (CT) (Figure A3). This advanced diagnostic examination was carried out on a multi-slice CT scanner (Sensation 16) with iodine contrast medium (370 mg/mL) at a dosage of 800 mg iodine/kg.

With the precise location of the intra-abdominal gonadal structures known, the surgical removal was planned and performed. There were no urethral injuries or other intra- or post-operative complications. Gross examination revealed right and left testicle-like structures attached to uterine horn-like structures. The patient was pre-medicated with Dexmedetomidine (Dexdomitor, Vétoquinol Italia S.r.l. (Forli, Italia), 2 mcrg/kg) and Methadone (Semfortan, Eurovet Animal Health B.V., Bladel, The Netherlands, 0.3 mg/mL), induced with Propofol (Propovet, Ecuphar Italia, 3 mg/kg) and maintained with Isoflurane (Isoflo, Zoetis Italia). After positioning the trichotomized patient in the dorsal recumbent decubitus position, a surgical scrub of the caudal abdominal and inguinal region was performed. The gonadal structures were identified and removed laparoscopically. Changing the decubitus to sternal and rearranging the sterile surgical field, the penile structure was removed. There were no urethral injuries or other intra- or post-operative complications. Gross examination revealed right and left testicle-like structures attached to uterine horn-like structures. The patient was followed up for 12 months after surgery. The dog did not develop a urinary tract infection or urinary incontinence for 12 months following surgery. Figure A4 shows the dog before surgery, at the end of the procedure, and after 6 months from the surgery with a complete healing of the external genitals.
