# Supplementary material for: Genomic Sequencing to Detect Cross-Breeding Quality in Dogs: An Example Studying Disorders in Sexual Development
Source: Int J Mol Sci. 2024 Oct 6;25(19):10763. doi: 10.3390/ijms251910763 (PMC11476854; doi:10.3390/ijms251910763)
Supplement: Supplementary file 1 [file ijms-25-10763-s001.zip › ijms-3183190-supplementary.pdf]

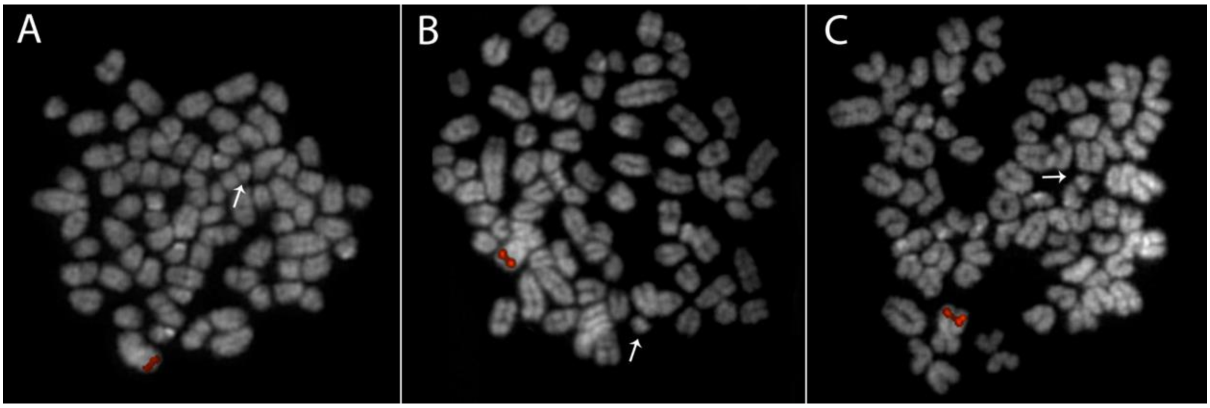

Figure S1. FISH analysis using an X-specific probe (CH82-201N14) in three healthy control dogs: (A) *Brutus*'s father, (B) *Bufalo*, and (B) *Tauro*. All three dogs exhibit a normal karyotype (78,XY), as indicated by the probe signal appearing on only one chromosome. Arrows point to the Y chromosomes in each panel.

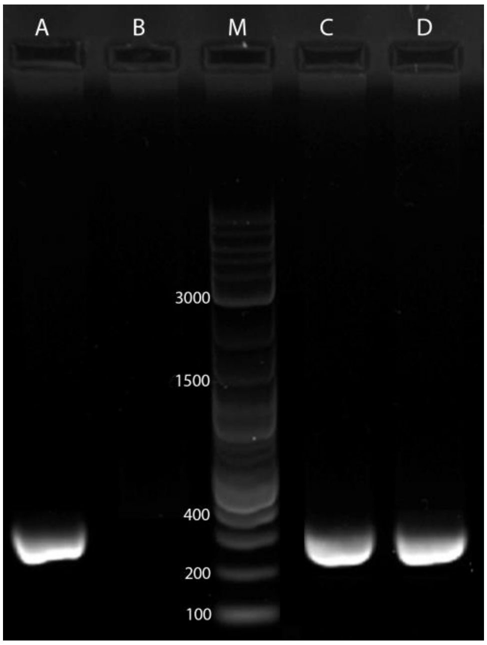

Figure S2. 1% agarose gel electrophoresis shows the PCR results performed with canine-specific SRY primers. Lanes are loaded as follows: *Brutus*'s father (A), *Brutus* (B), 2-log DNA marker (M; band sizes indicated in bp), *Bufalo* (C), and *Tauro* (D). The presence of the band in *Brutus*'s father, *Bufalo*, and *Tauro*, at the expected size (271 bp), indicates the presence of the SRY gene, while its absence in *Brutus* confirms the *SRY*-negative status.
